# Organ dose assessment in 15-year-old patients undergoing panoramic radiography: a Monte Carlo study using mesh-type phantoms and realistic modelling

**DOI:** 10.1007/s12194-026-01100-7

**Published:** 2026-07-20

**Authors:** Maria Rosangela Soares, Wilson Otto Batista, Jacqueline Machado Gurjão Rios

**Affiliations:** 1 Federal University of Rondônia, Porto Velho, Brazil; 2 Federal Institute of Bahia – Salvador Campus, Salvador, Brazil

**Keywords:** Monte Carlo simulation, Panoramic radiography, Mesh-type reference computational phantoms, Pediatric dosimetry, Organ dose

## Abstract

This study evaluated organ-specific absorbed doses and sex-dependent dosimetric variations in 15-year-old patients undergoing panoramic radiography across different units technologies. ICRP 156 mesh-type reference computational phantoms (MRCPs) were coupled with the PHITS Monte Carlo code for this analysis. Three panoramic units with distinct kinematics and exposure protocols were simulated: Unit A (fixed isocentric rotation, constant parameters) and Units B and C (sliding rotation axis, dynamic kV/mA modulation). Absolute absorbed doses were estimated using experimentally derived air kerma-area product conversion factors. Salivary glands and oral mucosa received the highest absorbed doses, ranging from 270 to 640 µGy, depending mainly on the tube voltage protocols. Sex-specific dosimetric disparities appeared due to anatomical variations interacting with beam geometry. The female MRCP absorbed 26% to 28% higher doses in the head lymph nodes due to smaller craniofacial dimensions, increasing scattered radiation contribution. Male phantoms systematically received 12% to 13% higher doses to the extrathoracic lymph nodes across all systems, due to a larger cervical circumference intersecting the divergent primary beam. Patient anatomy dictated the relative dose distribution, but the unit’s operational protocol, particularly dynamic modulation and beam energy, determined the absolute exposure magnitude. These findings emphasize that 15-year-old patients require age-specific protocol optimization. Using highly realistic MRCPs with dynamic exposure parameters shows that unit technology is the main determinant of patient risk, offering a framework for ALARA implementation in adolescent dental radiology.

## Introduction

Panoramic radiography is a fundamental two-dimensional imaging modality in contemporary dentistry, widely utilized due to its broad anatomical coverage and rapid acquisition [1–3]. Although panoramic radiography performed with modern digital units delivers effective doses typically in the range of 4–30 µSv [4, 5], its widespread use in adolescents still warrants careful dosimetric assessment. At approximately 15 years of age, patients undergo a high frequency of radiographic examinations, primarily driven by orthodontic planning and the evaluation of maxillofacial development. From a radiobiological perspective, adolescents exhibit higher cellular radiosensitivity and possess a longer life expectancy for radiation-induced stochastic effects to manifest compared to adults. Therefore, relying on generalized adult dosimetric models for these demographic underestimates the radiological risk. This transitional anatomical phase demands age-specific dose estimations to ensure that the diagnostic benefits clearly outweigh the risks, adhering to the ALARA (As Low As Reasonably Achievable) principle [6].

The assessment and estimation of exposure doses in both children (5–15 years) and adults have traditionally relied on anthropomorphic phantoms combined with Monte Carlo simulations, employing a variety of established codes [6–11]. Historically, organ dose assessments in radiological protection and medical imaging have relied on stylised and, more recently, voxel-based anthropomorphic computational phantoms [12]. Stylised mathematical phantoms allow flexible scaling of body size and posture, but they represent anatomy with simplified geometric shapes, which limits their ability to model small or thin structures such as the salivary glands and oral mucosa. Voxel phantoms provide a more realistic three-dimensional representation derived from patient images [13], but their discrete resolution restricts the accurate description of fine anatomical details and tissue interfaces, and changes in posture or anatomical adaptations are difficult to implement.

These inherent limitations motivated the development of mesh-type reference computational phantoms, which offer continuous organ surfaces, an improved description of thin tissues and blood content, and greater flexibility to adapt anatomy to specific exposure scenarios. Unlike voxel-based models, mesh geometries allow controlled anatomical deformation, posture adjustment and rescaling of organ size while preserving smooth organ boundaries and fine anatomical details [14, 15]. In this context, ICRP Publication 156 introduced a new generation of paediatric mesh-type reference computational phantoms, including 15-year-old male and female models that provide anatomically realistic representations of late adolescence and can be directly implemented in Monte Carlo codes without the need for auxiliary voxelisation tools.

Despite these methodological advances, dosimetric assessments in panoramic radiography for the 15-year-old age group have historically been extrapolated from adult data or focused on younger children aged 5–10 years [6, 8]. While recent studies have begun to address this specific age group [16], investigations employing mesh-type phantoms of high anatomical fidelity remain scarce, a gap that is particularly relevant given that adolescent organ positions and tissue compositions differ substantially from those of both children and adults, directly influencing radiation absorption patterns [14].

The existing literature identifies the salivary glands, oral mucosa, and thyroid as the organs receiving the highest doses during panoramic radiography, although values fluctuate depending on the specific unit and operational parameters adopted [4, 5, 9, 17]. The majority of current estimates are derived from physical anthropomorphic phantoms using point measurements—typically involving ionisation chambers, thermoluminescent dosimeters (TLD), Optically Stimulated Luminescence (OSL), or Metal-Oxide-Semiconductor Field-Effect Transistor (MOSFET) detectors [18, 19]. Notably, studies focusing on paediatric populations are considerably less common than those involving adults. When such studies are available, they typically report lower absolute doses in paediatric phantoms; however, this should be interpreted in light of the fact that the cancer risk associated with stochastic effects is substantially higher in the paediatric age group [17, 19–21].

Since the publication of ICRP 103 [22], the salivary glands and oral mucosa have been explicitly incorporated into dosimetric protocols. Particular attention is drawn to the salivary glands in this context: as they are directly exposed to the primary beam during panoramic radiography, they were assigned a specific tissue weighting factor (w_T_) to reflect their significance in effective dose calculation.

State-of-the-art panoramic unit has evolved significantly, incorporating advanced features such as tube voltage modulation during rotation and refined elliptical trajectories. Yet, these dynamic parameters are frequently simplified or entirely overlooked in many Monte Carlo simulations found in the literature [6, 8, 23].

Consequently, the central objective of this study is to bridge this gap by presenting precise dose estimates for organs and tissues in the head and neck region of 15-year-old patients, explicitly modelling the complex operational characteristics of modern radiographic systems. Specifically, absorbed doses were evaluated for dosimetrically relevant structures included in ICRP Publication 103 [24], namely the salivary glands, oral mucosa, thyroid, brain and extrathoracic airways. The analysis also quantified the influence of operational variables such as the centre of rotation (fixed versus sliding), kV modulation, and dose rate modulation.

## Materials and methods

**Fig. 1:**
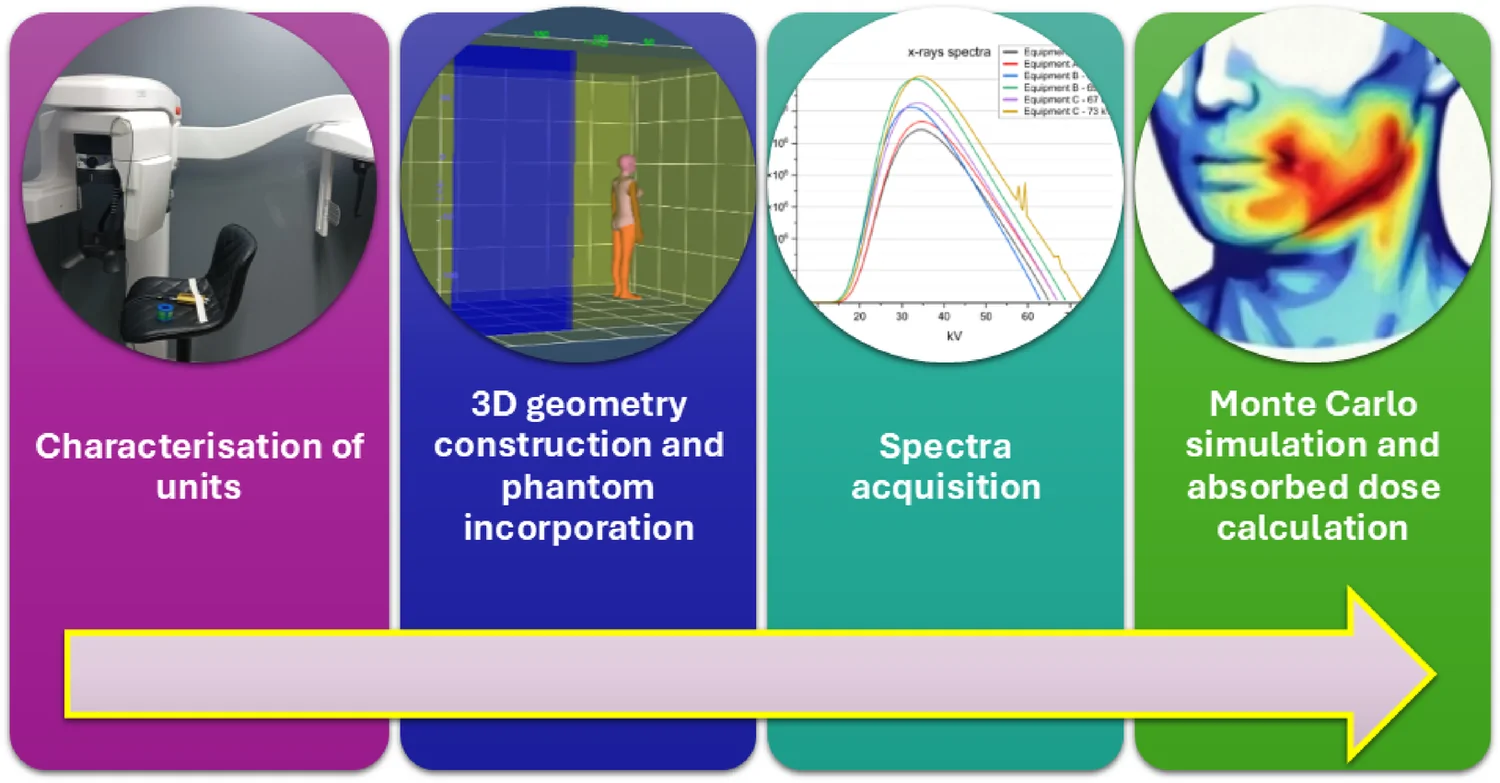
Representative diagram of the methodological workflow steps

To meet the proposed objective, this study adopted a structured methodology that seamlessly integrates the acquisition of experimental physical data with advanced computational simulations. The complete methodological workflow is illustrated in Fig. 1. The process advanced sequentially: beginning with the anatomical selection of the 15-year-old MRCPs and the spectral characterisation of the selected unit (preparation and configuration stages), progressing through the mathematical modelling of the X-ray beam trajectory, and culminating in the particle transport simulation and volumetric calculation of the absorbed dose. The following subsections detail the technical parameters and methodological choices that underpin each of these stages, starting with the anatomical core of the simulation.

### Units and its characteristics

Three dental panoramic radiography units were selected to meet the aims of this study, with particular attention to the differences in how each system modulates the X-ray beam. The key technical features considered were the methods of high-voltage (kV) modulation and dose-rate modulation, as these determine how the exposure is adjusted along the beam trajectory. In addition, one unit previously investigated in an evaluation of the rotation axis was intentionally included, enabling comparison with earlier work reported in the literature [3].

The units are identified by the letters A, B and C: (A) Dabi Atlante/Eagle, operating with a beam without tube-voltage (kV) or dose-rate modulation; (B) Instrumentarium/OP300 and (C) Vatech/PaX, both employing modulation of kV and dose rate during rotation. The information required for Monte Carlo modelling of these systems, including geometric setup and exposure parameters, is summarised in Table 1.

**Table 1:** Technical specifications for Monte Carlo simulation of the three units studied

Parametrs	Unit A	Unit B	Unit C
Maximum kV and mA	90/12.5	90/16	90/10
Beam width and height (cm)*	0.64/15	0.6/14.3	0.6/15.04
Total filtration (mm of Al)	3.25	2.5	2.8
Distance focus-object (cm)	40	39.8	37.5

*Values at the image receptor surface

The dosimetric assessment and waveform acquisition for the three units were carried out using the *Radcal AccuGold* system [25], which enables simultaneous dose measurements and detailed recording of the exposure signal. With the sensor placed on the image receptor surface, data were collected using two protocols available for the patient’s body type, corresponding in this study to 15-year-old male and female models. For each unit, the pre-programmed protocols that best matched these biotypes were selected, ensuring that the exposure profiles reflected clinically representative operating conditions.

### Monte Carlo simulation set-up

#### PHITS code

The Particle and Heavy-Ion Transport Code System (PHITS) [26] is a versatile, general-purpose Monte Carlo particle transport code, developed primarily by the Japan Atomic Energy Agency (JAEA) to simulate particle interactions in complex geometries. It is widely applied across multiple scientific fields, particularly in medical physics, because it can model the transport of photons, electrons, neutrons, and heavy ions over a broad energy range, from around 10⁻⁵ eV up to the TeV region, depending on the particle type and selected physics models. The combination of comprehensive physics models and flexible geometry handling makes PHITS a valuable tool for medical applications such as dental radiology, patient dose assessment, and the optimisation of radiological protection strategies.

To address the challenges associated with double-defined regions at the interface between the unit’s Constructive Solid Geometry and the phantoms’ mesh geometry, which arise from their relative dimensions and positioning, this study adopted a two-step approach. Firstly, dump files were generated immediately downstream of the collimation system and subsequently utilised as the radiation source in the simulations. PHITS dump files store intermediate particle information (such as position, direction, and energy), enabling their reuse in repetitive simulations. This strategy substantially reduces computation time by allowing for modifications to the geometry or source parameters without the need to re-evaluate unchanged particle histories, thereby significantly optimising iterative analysis and overall simulation efficiency.

#### Generation of X-ray emission spectra

**Fig. 2:**
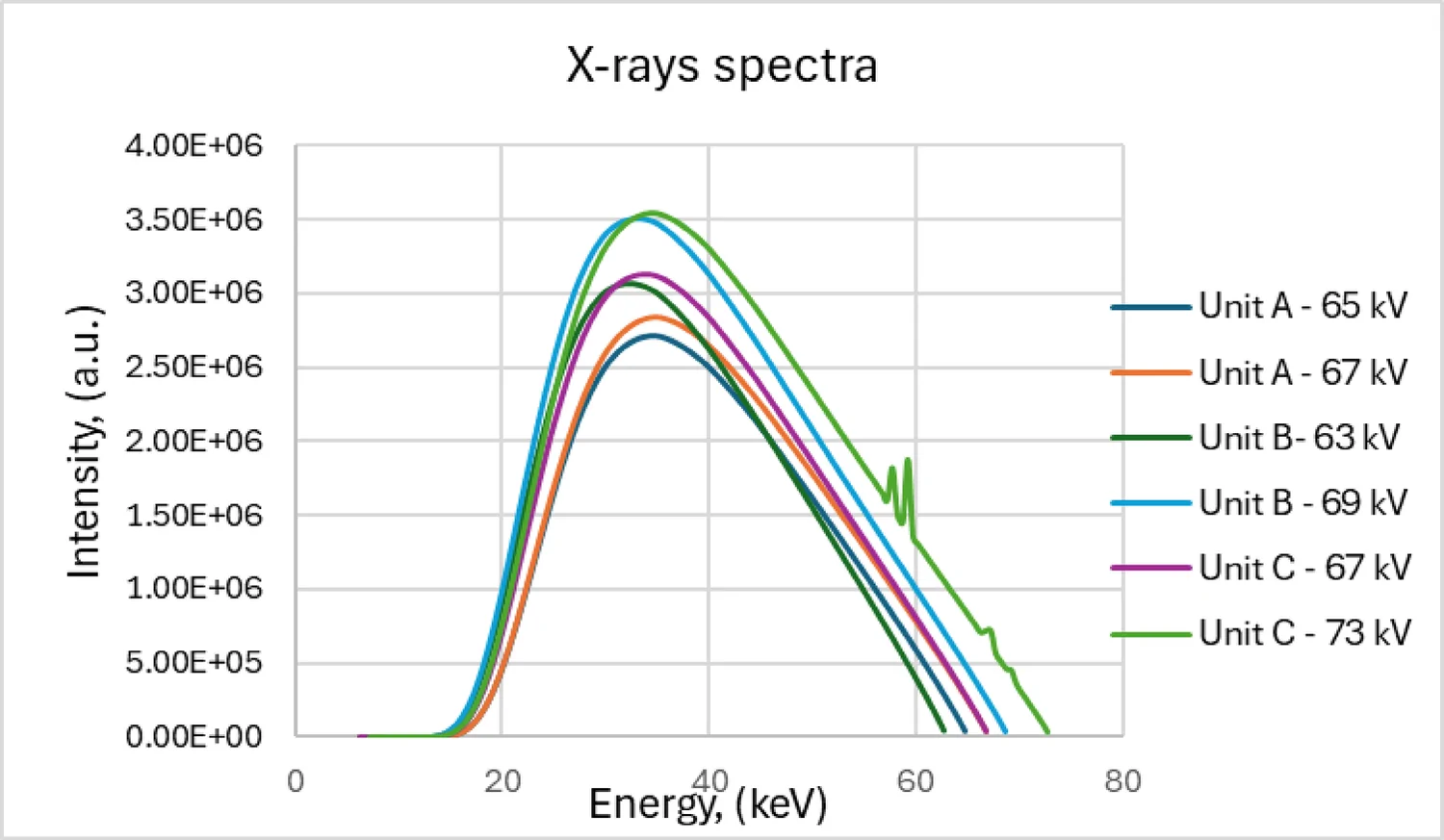
Emission spectra of the X-ray tubes used, generated with the aid of the SpekPy Web (version 2.0.8) [27]

X‑ray emission spectra were generated in 1 kV increments using SpekPy Web (version 2.0.8) [27]. covering the tube potential range used in the panoramic protocols. All three units employ Toshiba/Canon X‑ray tubes (models D‑054SB or D‑051SB) [28], so only the total filtration specific to each unit needed to be adjusted in SpekPy (Table 2). For each combination of tube potential and filtration, SpekPy Web was used to calculate the corresponding tungsten-anode spectra, which were exported as energy-fluence tables. These spectra were then normalised and implemented in PHITS as the photon source term for the simulation of the X‑ray tube and collimation system, as indicated in the Spectra generation (Fig. 2) step of the methodological workflow.

**Table 2:** Parameters used in SpekPy Web (version 2.0.8) for X-ray spectrum generation

Unit	Tube potentials used (kV)	Total filtration (mm Al eq.)	Anode material	Anode angle (°)
A	65, 67	3.25	W	5
B	63, 69	2.50	W	5
C	67, 73	2.80	W	5

The tube potential, total filtration and anode characteristics were defined in SpekPy Web (version 2.0.8) to generate the X ray spectra, while the focus–object distance for each unit (Table 2) was modelled explicitly in the PHITS geometry. To obtain the spectra using the Web SpekPy tool, we set the parameters as follows: minimum energy at 10% of the maximum value; 1 keV energy steps; CASIM physics model; and PENELOPE-based attenuation data. Values for filtration, distances, anode angle, and other technical parameters were extracted from the unit technical manuals [27].

#### Fitting of the rotation axis curve/trajectory

The rotation axis trajectory was modelled as an elliptical path composed of two branches of an ellipse. The curve describing this trajectory was fitted using a Cauchy function (Eq. 1), following the approach described by Byung-Ju Joh et al. (2024) [3].

1 $$\:y=\frac{1}{\left(a\times\:{\left(x+b\right)}^{2}+c\right)}+d$$

with the fitting parameters had the following values: * a=0.01033*, * b=4.4620*, * c=-0.35950*, and * d=-32.900*, * -32.700*, and * -30.420* for unit A, B, and C, respectively. This additive constant * d* is used to reposition the coordinate system. The curve fitting was performed using the LabFit software developed at the Federal University of Campina Grande [29].

**Fig. 3:**
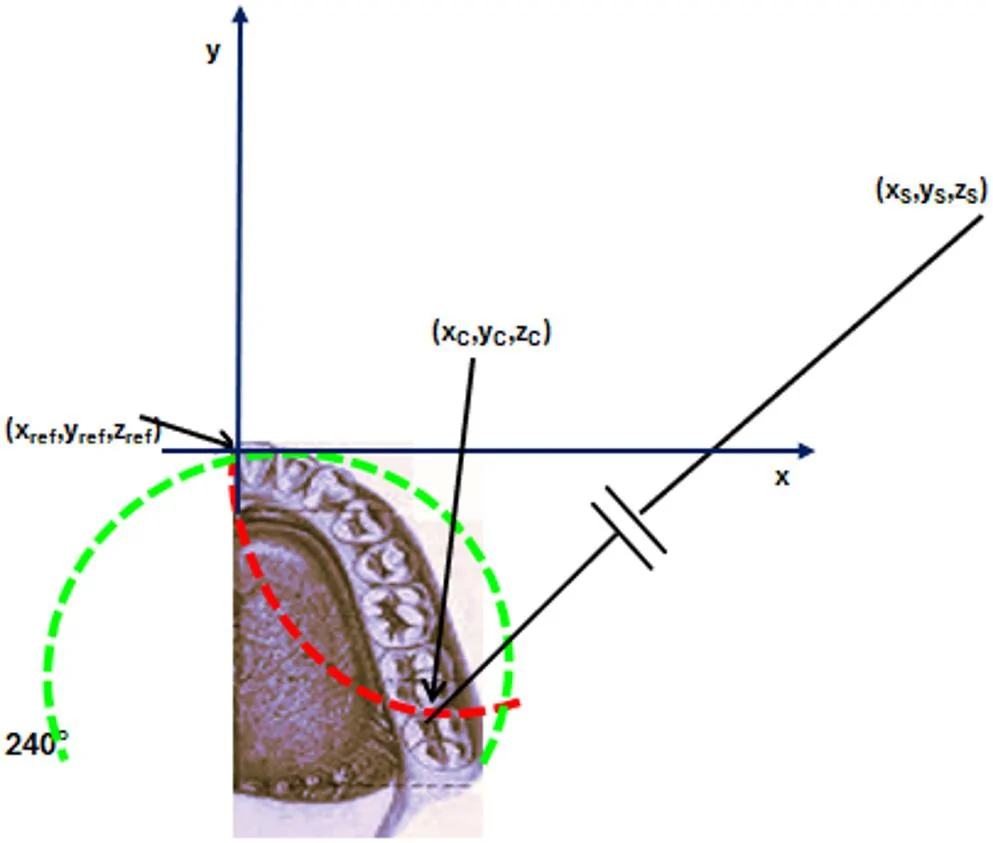
Representation of the reference system used for positioning the phantom and the x-ray source. The points (x_ref_, y_ref_, z_ref_); (x_C_, y_C_, z_C_) and (x_**S**_, y_**S**_, z_S_) correspond to the coordinates of the reference point, the rotation axis, and the X-ray source position, respectively. The rotation axis trajectory is shown in red

The fitted curve, together with the reference coordinate system adopted to position the phantoms relative to the X-ray source, is presented in Fig. 3.

The reference point was defined according to the patient positioning landmark used for panoramic radiograph acquisition, corresponding to the occlusal contact of the anterior teeth with the bite block. In the simulations, the X-ray sources are represented by Dump Files, which are placed in the geometry 14 cm away from this point (x_s_, y_s_, z_s_).

#### Source setup

The simulations were performed using PHITS with dump files containing 1.0 × 10⁸ particles each, generated for each tube voltage (kV) value employed in the modulation protocol and assigned to their corresponding angular positions along the 240° rotational arc. A multi-source file comprising 161 sources spaced at 1.5° intervals was constructed to cover the full exposure trajectory [7], yielding a total of 1.61 × 10¹⁰ particles per simulation scenario. To enable accurate rotational modelling, a separate file containing 161 coordinate transformations, formatted according to the specific PHITS syntax [26], was implemented. Each dump file, with an average size of approximately 300 GB, stores for every particle the spatial coordinates, propagation direction, kinetic energy, and statistical weight associated with each energy interval [30]. This approach permits reuse of the beam phase space already transported to the collimator exit, thereby avoiding inconsistencies arising from overlapping or double-defined geometries and eliminating the need to recalculate the initial particle transport for each angular position or kV setting, which significantly enhances computational efficiency while preserving dosimetric fidelity.

In the PHITS code, coordinate transformations are configured to execute spatial translation prior to geometric rotation. For this study, we adopted the M = − 3 mode, where the transformation input follows the syntax: $\:\mathrm{T}\mathrm{R}\mathrm{n}\:\mathrm{X}0\:\mathrm{Y}0\:\mathrm{Z}0\:\mathrm{X}\mathrm{C}\:\mathrm{Y}\mathrm{C}\:\mathrm{Z}\mathrm{C}\:\mathrm{A}1\:{\uptheta\:}1\:\mathrm{A}2\:{\uptheta\:}2\:\mathrm{A}3\:{\uptheta\:}3-3$. In this syntax, $\:{X}_{0}$, $\:{Y}_{0},$ $\:{Z}_{0}$ and specify the spatial components of the translation along the * x*, * y* and * z*-axes define the coordinates of the centre of rotation along these same axes. To ensure the precise movement of the source $\:{X}_{C}$, $\:{Y}_{C}$ and $\:{Z}_{C}$ establish the coordinates for the centre of rotation across these same axes. The sequence $\:{A}_{i}\:$and $\:{\theta\:}_{i}$($\:\mathrm{w}\mathrm{h}\mathrm{e}\mathrm{r}\mathrm{e}\:i=\mathrm{1,2},3$) defines the target axis and the angle of the * i*-th rotation, respectively. Values of $\:{A}_{i}\:$equal to 1, 2, or 3 correspond to rotations applied around the * x*-, * y*-, and * z*-axes. The computational files containing all specific parameters for each transformation can be made available upon request.

#### Simulated protocols

A total of twelve scenarios were simulated, combining two kV settings, beam modulation and a sliding rotation axis, as summarised in Table 3. These scenarios were configured to incorporate both the sliding axis and radiation beam modulation (kV and dose rate).

**Table 3:** Identification of the sixteen protocols and thirty-two scenarios (Male/Female)

No/Scenario		kV	Sliding axis	Modulation
1	1 A - M/F	65	Yes	No
2	2 A- M/F	67	Yes	No
3	1B- M/F	63	Yes	Yes
4	2B- M/F	69	Yes	Yes
5	1 C- M/F	67	Yes	Yes
6	2 C- M/F	73	Yes	Yes

The scenarios comprise two kV settings, modulation of kV and dose rate, and either a sliding or fixed rotation axis. The units are identified by letters. The scenarios are identified by a number, a letter indicating the unit, and a letter denoting the phantom’s gender

*M* Male, *F* Female, *Modulation* modulation in x-ray emission kV and/or dose rate

#### kV waveform and dose rate

**Fig. 4:**
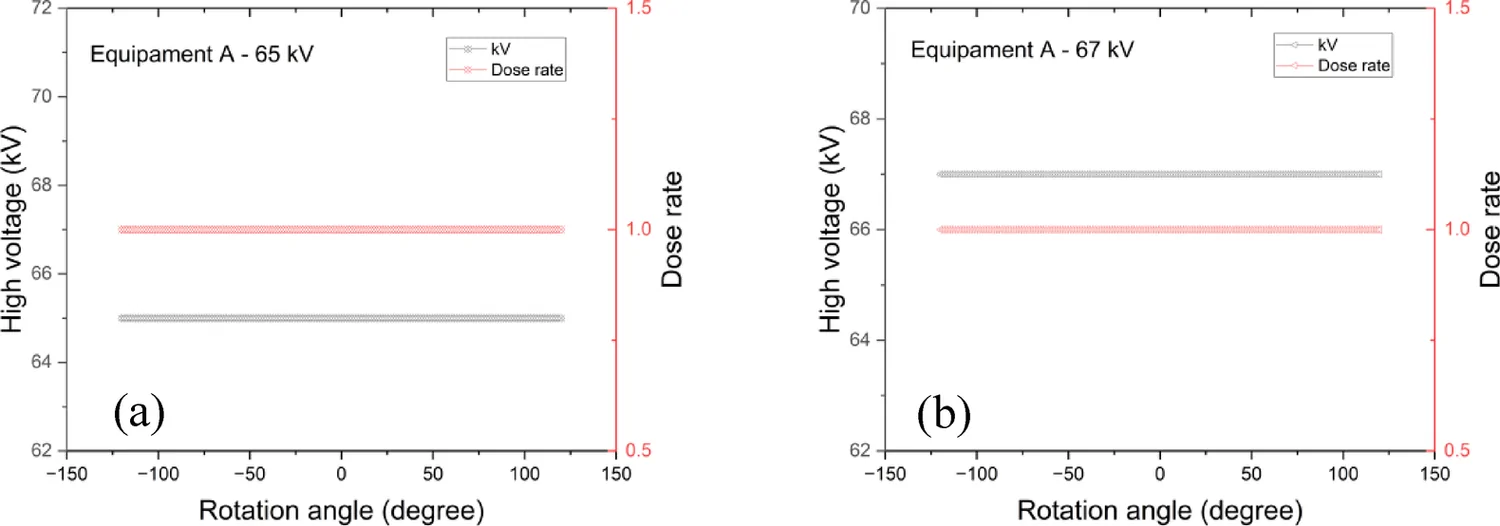
Waveform assessed for Unit A operating at **a** 65 kV and **b** 67 kV, plotted as a function of the angular position of the tube–detector system over the full 240° rotation arc (− 120° to + 120°). The dose rate is normalised to unity at the initial value. Unit A operates without kV or dose-rate modulation; both parameters remain constant throughout the entire exposure arc

**Fig. 5:**
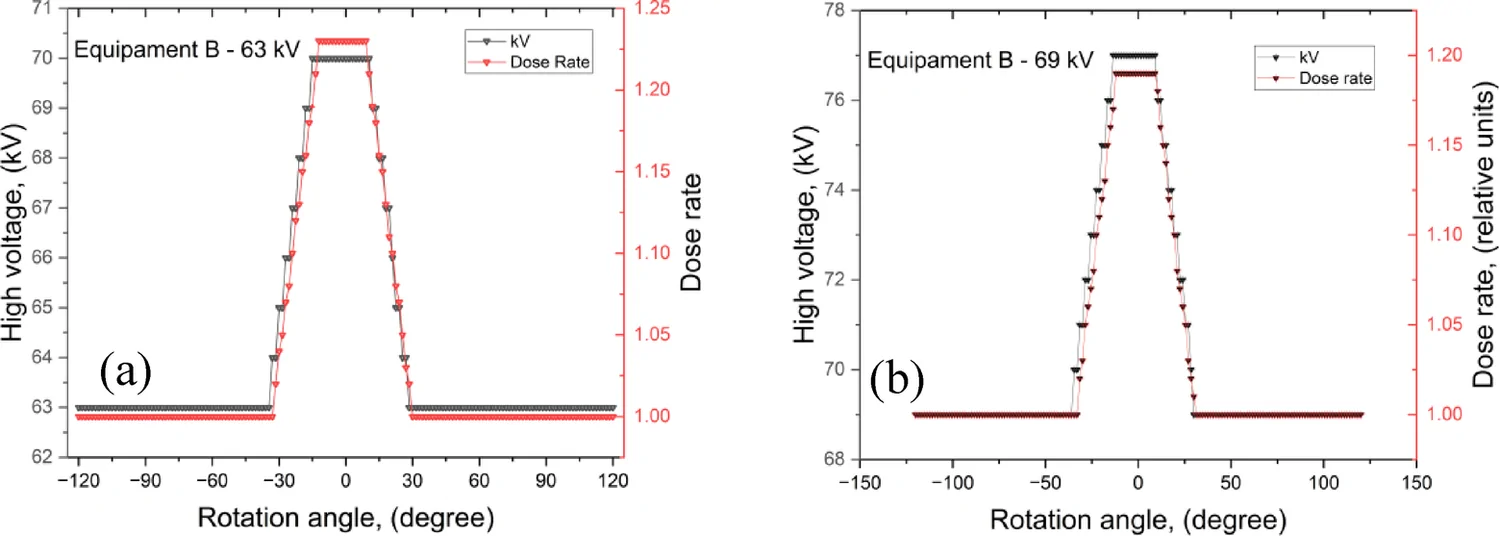
Waveform assessed for Unit B operating at **a** 63 kV and **b** 69 kV, plotted as a function of the angular position of the tube–detector system over the full 240° rotation arc (− 120° to + 120°). The dose rate is normalised to unity at the initial value. Both kV and dose rate are dynamically modulated within a restricted angular sector (approximately − 30° to + 30°) to compensate for the increased beam attenuation through the posterior cervical spine; the tube voltage rises from 63 to approximately 70 kV in protocol (**a**) and from 69 to approximately 77 kV in protocol (**b**)

**Fig. 6:**
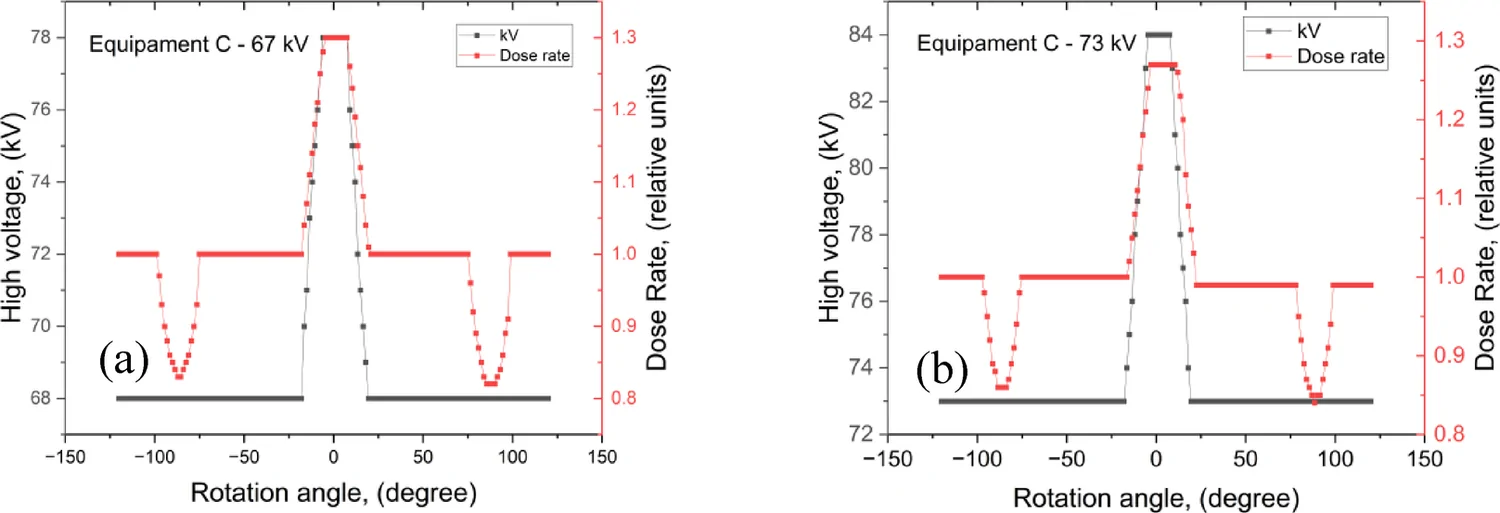
Waveform assessed for Unit C operating at **a** 67 kV and **b** 73 kV, plotted as a function of the angular position of the tube–detector system over the full 240° rotation arc (− 120° to + 120°). The dose rate is normalised to unity at the initial value. Both kV and dose rate are dynamically modulated within a restricted angular sector (approximately − 20° to + 20°) to compensate for beam attenuation through the posterior cervical spine; the tube voltage rises from 68 to approximately 78 kV in protocol (**a**) and from 73 to approximately 84 kV in protocol (**b**). Additionally, Unit C applies dose-rate modulation over a wider lateral arc to compensate for facial attenuation

The tube voltage (kV) and dose‑rate waveforms of the three panoramic radiography units were recorded experimentally for all exposure protocols. Both signals were sampled continuously over the entire exposure time of each rotational acquisition and initially plotted as a function of time. The measured kV and dose rate were then mapped to the angular position of the tube–detector system so that the interval from − 120° to 120° represents the full 240° rotation arc, and the resulting angle‑dependent kV and dose‑rate functions were implemented as the source description in the Monte Carlo simulations. For each unit, the kV waveform and the corresponding dose rate are displayed in Figs. 4, 5 and 6, with the dose rate normalised to unity at its initial value to allow direct comparison between protocols and units.

#### Geometry construction

**Fig. 7:**
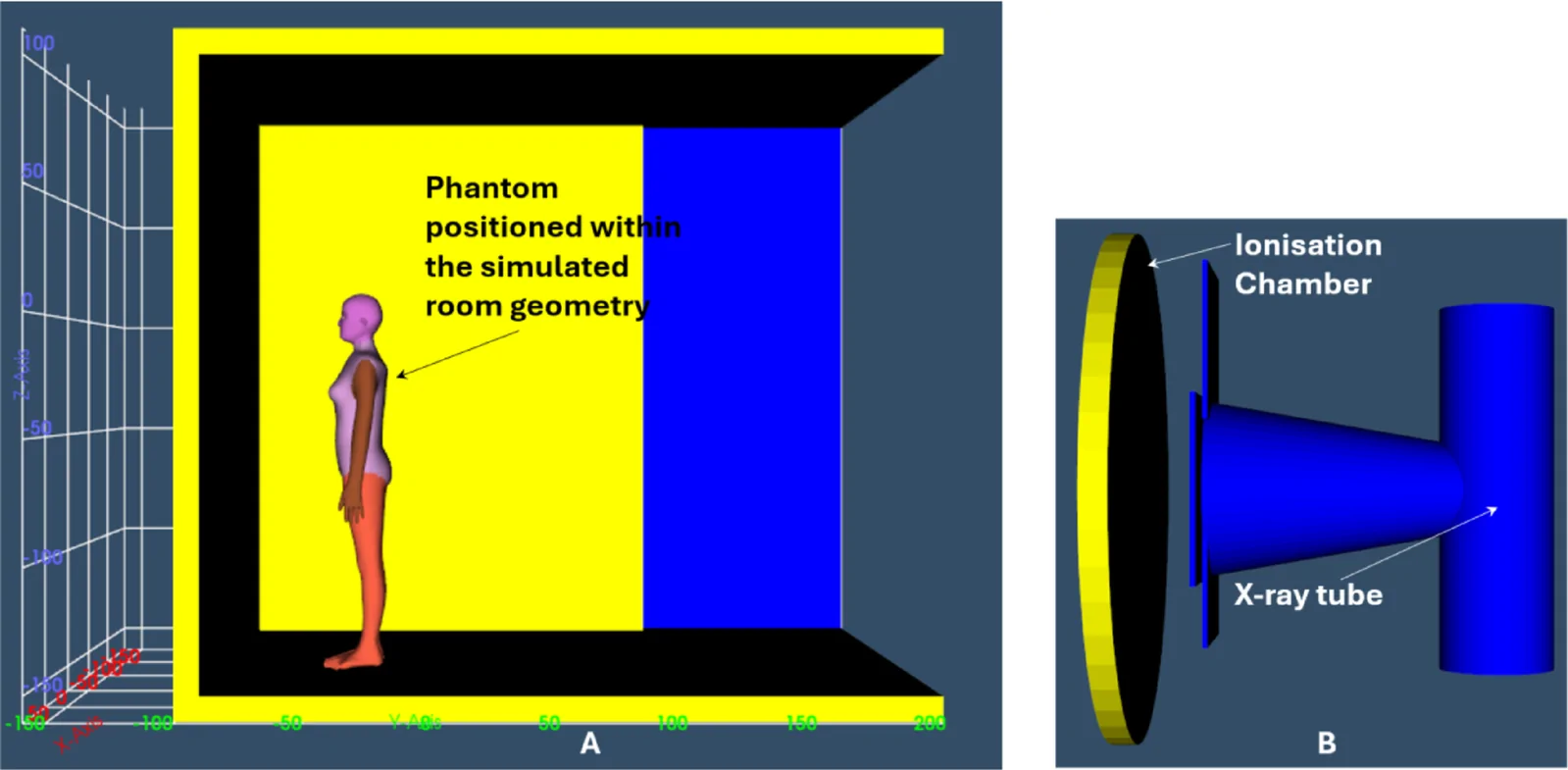
**A** Representation of the three-dimensional room geometry illustrating the phantom positioning for the simulations. **B** Details of the X-ray tube and collimation system. Representation of the ionisation chamber used for determination of the simulated air kerma-area product

The geometry used in this study comprises a room with typical dimensions for dental panoramic radiography examinations, together with the structural model of the X-ray tube derived from Toshiba/Canon technical manuals [28]. The room (Fig. 7a) was modelled with a length of 3.30 m, a width of 2.50 m, and a height of 2.50 m. The walls, floor, and ceiling were represented as 5 cm thick concrete, and the door was modelled with an equivalent lead thickness of 0.05 cm. All geometry in the Constructive Solid Geometry (CGS) system was constructed using the *SimpleGeo* software, which serves as a solid modeller for radiation transport simulations [31].

The X-ray tube (Fig. 7b) was modelled as a lead housing consisting of a cylinder with a diameter of 4.54 cm and a wall thickness of 0.2 cm. The collimation system comprises a cone and lead plates, each with a thickness of 0.2 cm, which define the useful beam emitted by the unit. Inside the 4.54 cm lead cylinder, a vacuum region was assumed together with a tungsten cylinder whose face is cut at a 5-degree angle, representing the anode target of the X-ray tube [28].

### Absorbed dose estimation

#### The ICRP-MRCP phantoms

ICRP Publication 156 presents a series of paediatric reference computational phantoms based on mesh-type geometry, designed to provide a flexible and anatomically realistic representation of children from birth up to 15 years of age [15]. Within this set, the 15-year-old male and female reference phantoms play a particularly important role, as they capture key anatomical features of late adolescence, including organ size and position, overall body proportions, and tissue composition.

The specification data for the 15-year-old ICRP-MRCP male and female phantoms are presented in Table 4.

**Table 4:** Specifications of the 15-year-old ICRP-MRCP phantoms [15]

Parameters	Anatomical parameters	
	Male	Female
Height (cm)	167	161
Total body mass (kg)	56	53
Number of tetrahedra	7.366.440	7.519.627
File size - ASCII (MB)	412	422
Average tetrahedron volume (mm³)	7.43	6.96

##### Derivation of conversion factors

**Fig. 8:**
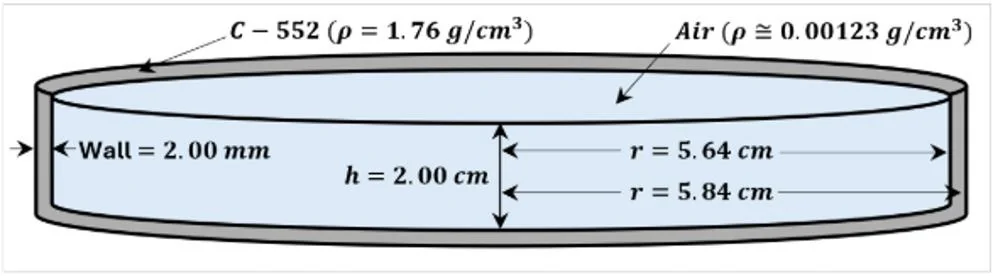
Schematic longitudinal cross-section of the ionisation chamber model implemented in PHITS for air kerma–area product estimation. The sensitive volume (radius 5.64 cm, height 2.00 cm) is filled with air at standard density (ρ = 0.00123 g/cm³) and enclosed by a 2 mm thick wall of C-552 air-equivalent plastic (ρ = 1.76 g/cm³)

For each computationally modelled panoramic radiography unit, a virtual ionisation chamber was incorporated to estimate the simulated air kerma–area product. This chamber was constructed from air-equivalent C-552 [30, 32] plastic, with 2.0 mm thick walls. The internal volume of 200 cm³ (radius 5.64 cm, height 2.00 cm) was filled with ambient air, centred on the beam axis and positioned at the collimator exit. A schematic representation of the chamber model is shown in Fig. 8.

The simulated air kerma–area product is the sum of all 161 beams and is presented in Eq. 2.

2 $$\:{P}_{KA,MC}=\sum\:_{i=1}^{161}{P}_{KA,MC,i}\left(kV\right)\times\:{w}_{i};$$

where P_KA, MC_ is the air kerma–area product simulated by the Monte Carlo method, and kV and wi are the tube voltage and the weighting factor representing the dose rate contribution of each source, respectively.

Electron, positron, and photon transport within the PHITS code was simulated using the EGS5 algorithm, activated by setting the parameter negs = 1. The energy cut-off thresholds were set to the default values recommended in the PHITS manual for EGS5: emin(12,13) = 0.1 MeV for electrons and positrons, and emin(14) = 0.001 MeV for photons. Since the maximum tube potential evaluated was 73 kV, all secondary electrons generated by photon interactions have energies below this threshold and their residual energy is deposited locally at the point of interaction. Cross-section data were retrieved from the standard PHITS data library. The absorbed dose in the regions of interest was scored using the energy-deposition tally method [26].

Statistical uncertainties are reported in the PHITS code as relative errors [26]. To obtain the simulated P_KA_ values, five simulation runs were performed for each kV setting.

#### Calculation of absorbed dose

In PHITS, the absorbed dose is directly obtained in units of Gy per source particle using the T-Deposit tally, provided that the mass, volume, and density of each region are correctly specified in the input file. The clinically relevant absorbed dose was then estimated from the ratio between the nominal value air kerma at the beam output and the air kerma calculated by the code, applying this conversion factor to the per-particle dose values yielded by PHITS (Eq. 3) [26].

3 $$\:{D}_{abs}={D}_{PHITS}\times\:\frac{{P}_{KA,nominal}}{{P}_{KA,PHITS}}$$

where $\:{D}_{\mathrm{abs}}$ is the absorbed dose in the region of interest, $\:{D}_{\mathrm{PHITS}}$ is the per-particle dose obtained from the T-Deposit tally, $\:{P}_{KA,nominal}$ is the experimentally measured air kerma at the beam output, and $\:{P}_{KA,PHITS}$ is the air kerma calculated by the code for the same geometric and spectral configuration.

The ratio ($\:{P}_{KA,nominal}/{P}_{KA,PHITS})$ in Eq. (3) acts as an absorbed dose calibration factor. The PHITS T-Deposit tally yields dose per source particle, a normalised quantity with no direct correspondence to a real clinical exposure. To convert this into a physically meaningful absorbed dose, it must be scaled by a factor that reflects the actual photon fluence delivered by the unit. The air kerma–area product was chosen as the scaling reference because it is directly measurable at the beam output and is proportional to the total energy deposited by the beam per unit area per exposure. The ratio therefore provides the multiplicative scaling factor required to convert the per-particle simulated dose into the absorbed dose corresponding to the actual clinical exposure, preserving consistency between the geometric and spectral conditions of the simulation and the experiment.

Our input dose-rate data were normalised to unity at the start of the exposure. As a result, the simulated $\:{P}_{KA,PHITS}$ does not carry the absolute intensity of the clinical beam, and the conversion factors in Table 5 therefore differ substantially from unity, ranging from 3.88 to 9.38. Unit A, which operates without kV or dose-rate modulation, produced the lowest and most stable conversion factors (3.88–3.96), indicating strong agreement between the simulated spectral output and the measured beam intensity. In contrast, the higher factors for Units B and C arise from their use of dynamic kV and dose-rate modulation: the nominal value $\:{P}_{KA,\mathrm{nominal}}$ integrates the total energy delivered over the entire exposure arc, including modulation peaks, whereas the simulation assumes a uniform source intensity distributed across the 161 angular positions. The notably high factor of 9.38 for Unit C at 73 kV aligns with its pronounced dose-rate modulation at peak voltage, which disproportionately increases the experimental $\:{P}_{KA,nominal}$ compared to the uniform-intensity simulation model.

**Table 5:** Multiplicative conversion factors ($$\:{P}_{KA,nominal}$$$$/{P}_{KA,PHITS}$$) applied to normalize the PHITS Monte Carlo output into absolute absorbed dose for the evaluated panoramic radiography units

Units	Tube voltage (kV)	Conversion factor
Unit A	65	3.96
	67	3.88
Unit B	63	5.03
	69	5.68
Unit C	67	5.18
	73	9.38

##  Results and discussion

The following sections present and discuss the absorbed dose estimates obtained for the head-and-neck organs and tissues of the 15-year-old male and female MRCPs across the three panoramic units evaluated, analysing the influence of sex, tube voltage, and beam modulation on the dosimetric outcomes. The results are also compared and discussed in the context of experimental dosimetric studies available in the literature.

### Absorbed dose in the head and neck: effect of sex and kV modulation in units A, B and C

**Fig. 9:**
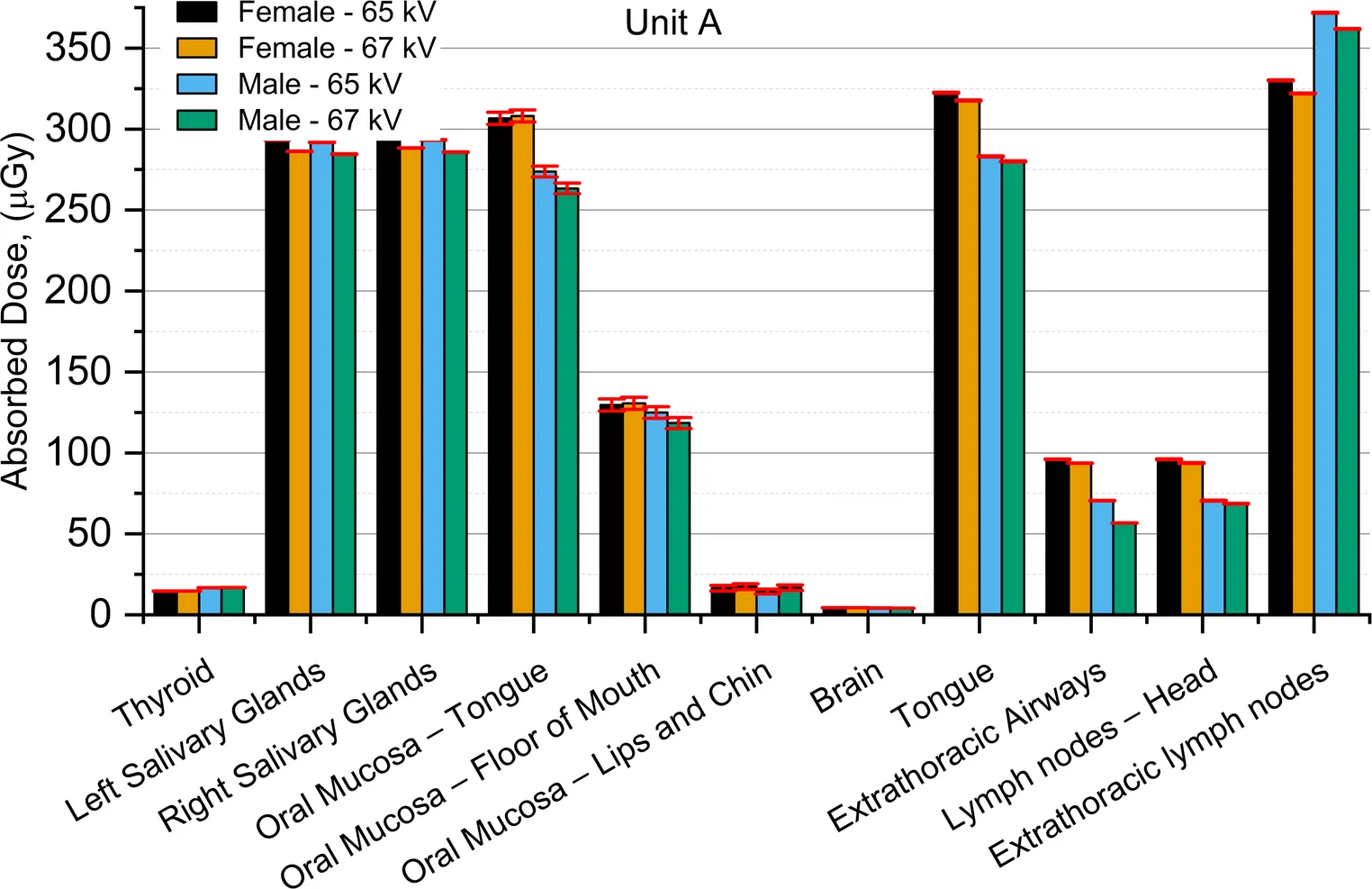
Absorbed dose distribution in head-and-neck tissues and organs for unit A, shown separately for the female and male phantoms. The error bars are shown in red and represent the relative error. The relative error values fall within the range of [0.05% – 10.60%]

**Fig. 10:**
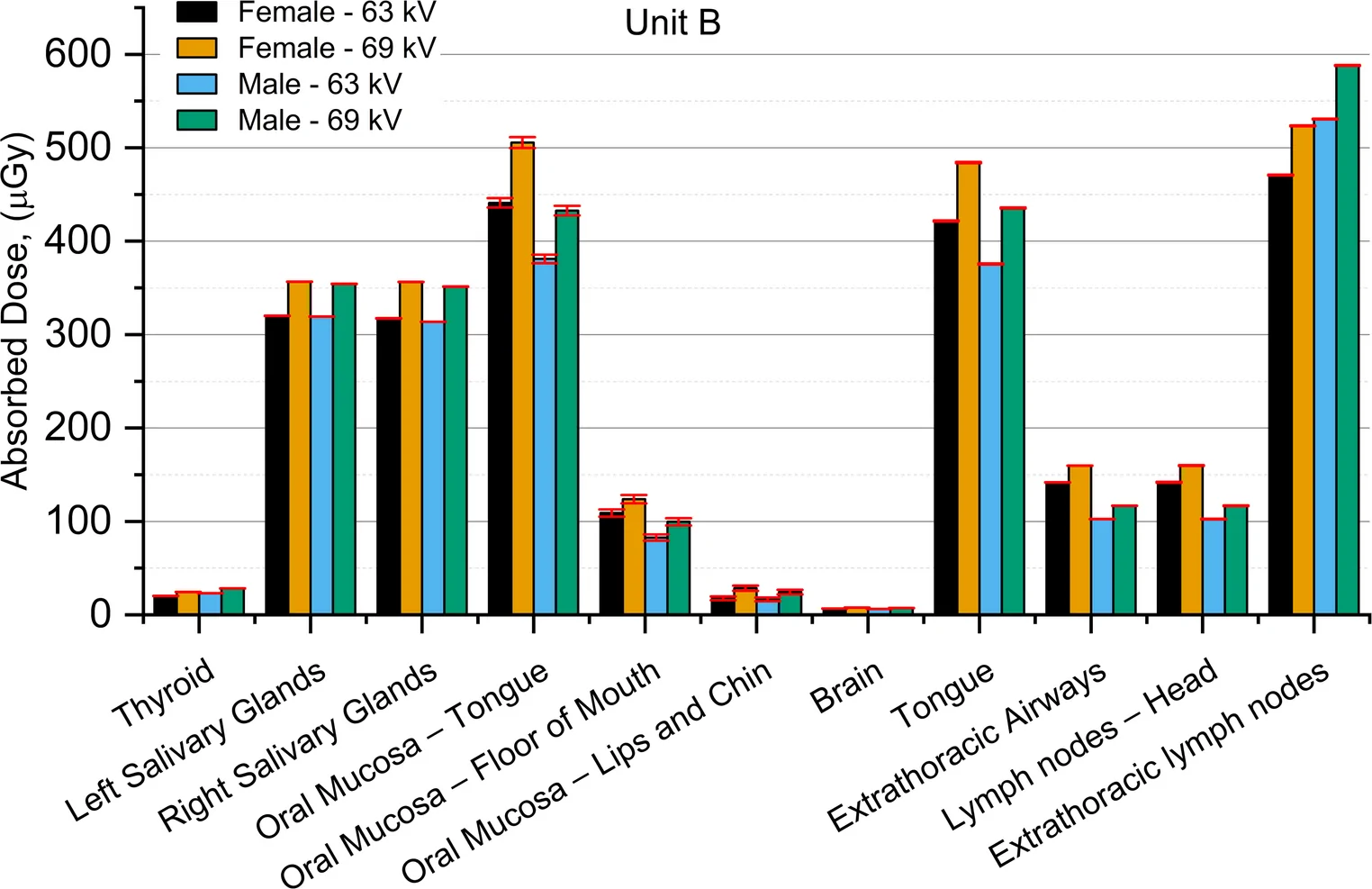
Absorbed dose distribution in head-and-neck tissues and organs for unit B, shown separately for the female and male phantoms. The error bars are shown in red and represent the relative error. The relative error values fall within the range of [0.06% – 11.72%]

**Fig. 11:**
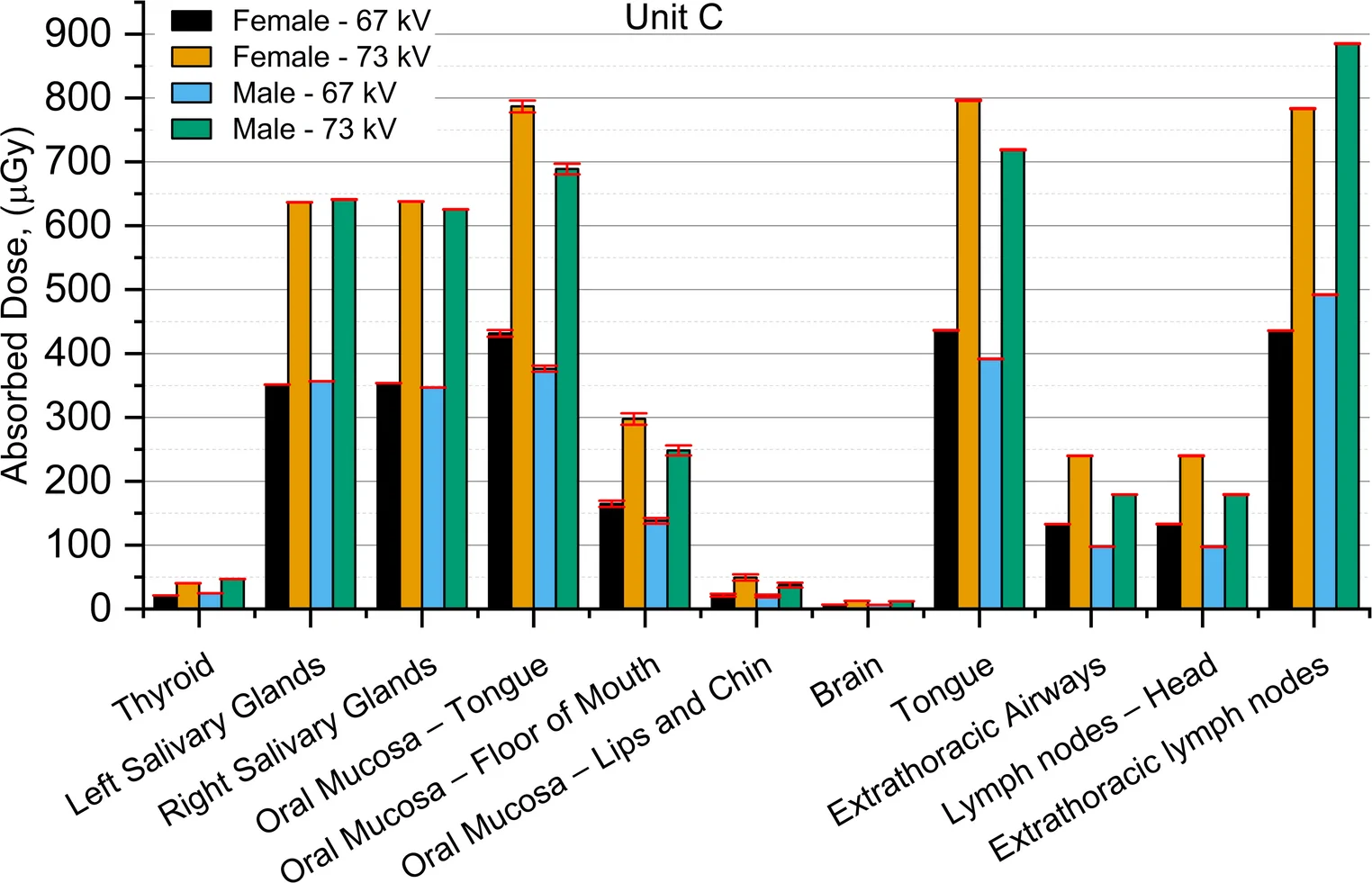
Absorbed dose distribution in head-and-neck tissues and organs for unit C, shown separately for the female and male phantoms. The error bars are shown in red and represent the relative error. The relative error values fall within the range of [0.06% – 10.88%]

The results of these simulations are detailed below. Figures 9, 10 and 11 illustrate the absorbed dose distributions in head-and-neck tissues for the three panoramic units evaluated (A, B and C, respectively). In each figure, data are stratified by sex to highlight the variations in absorbed dose to organs with respect to gender. A Relative percentage difference in absorbed dose: male regarding female,$$\:\:\%Ratio=$$$$\:\left({Dose}_{Male}-{Dose}_{Female}/{Dose}_{Female}\right)$$ is presented in the Table 6. The female phantom was adopted as the reference value in the ratio, following the convention used in sex-comparative dosimetric studies, in which the female model serves as the baseline given that it generally receives higher absorbed doses in cervical organs due to smaller craniofacial dimensions and closer proximity of critical structures to the primary beam.

The error bars in Figs. 9, 10 and 11 are shown in red and represent the relative error for the dose assessment in each organ/tissue, provided directly in the PHITS code results. The relative error values obtained in this study fall within the range of (0.05% − 11.72%).

### Absorbed dose in tissues and organs in units A, B and C

#### Thyroid

In all units, a consistently higher of absorbed dose was observed in the thyroid of the male phantom, for the same energy settings, with an average increase of 14–16% compared with the female phantom (Table 6; Figs. 9, 10 and 11). Although the thyroid is not the target organ of the examination and receives predominantly scattered radiation, this asymmetry between sexes is associated with subtle anatomical differences. According to ICRP Publication 156 [15], the thyroid of the adolescent male phantom presents a larger functional volume and a blood content approximately 36% higher than that of the female (2.04 g vs. 1.50 g), despite a similar total glandular mass. This increase in effective volume favours greater interception and attenuation of scattered radiation, resulting in a higher mean dose in the male phantom.

This trend is not frequently observed in experimental studies using TLDs positioned in physical PMMA or resin phantoms [4, 11, 33], nor in studies with stylised or voxel phantoms [6, 34], in which cervical anatomy is simplified and vascularisation is not represented. In addition, the point-based reading of TLD-100, combined with positioning uncertainties, increases the variability of the results and may mask subtle differences that become evident in volumetric approaches with mesh-type phantoms. In the typical high voltage range of panoramic examinations (60–80 kV), where the photoelectric effect still contributes significantly, small anatomical and tissue composition differences can intensify absorption in the cervical region, reinforcing the higher dose observed in the male phantom [4].

#### Tongue and oral mucosa

In contrast to the thyroid, the tongue showed systematically higher doses in the female phantom, with increments of 9–18% relative to the male phantom (Table 6). This behaviour can be explained by external shielding and volume effect. The so-called “mandibular shielding effect” is more pronounced in the male phantom, whose craniofacial architecture exhibits greater bone and muscle thickness, attenuating the beam before it reaches the intraoral tissues; the smaller mandibular thickness in the female phantom reduces this protection, increasing photon fluence within the oral cavity [36]. Additionally, anatomical differences in organ mass contribute to this pattern: according to ICRP Publication 156 [9], the male tongue has a slightly greater mass than the female tongue (56 g vs. 53 g).

The oral mucosa follows the same dosimetric pattern, as it is located in the same anatomical region and shares the same scenario of bony shielding and exposure to scattered radiation.

#### Salivary glands

In contrast to what is observed for the tongue and oral mucosa, where bony shielding and volume effects favour one sex, these factors act in a more balanced manner in the salivary glands. The salivary glands therefore showed a dosimetric equilibrium between sexes, with mean relative variations of approximately ± 1.5% in the absorbed dose of the female phantom compared with the male (Table 6). A plausible explanation is the more superficial anatomical position of the parotid glands, partially outside the protection of the mandibular cortical bone that shields the tongue. In these structures, there is a competition between two opposing effects: greater external attenuation in the male neck, due to increased muscle mass, and higher intra-organ blood content. This combination tends to cancel out, resulting in similar mean doses in the male and female phantoms [35].

Furthermore, regarding the absolute absorbed dose in the salivary glands, values ranged significantly from 270 µGy to 640 µGy across the evaluated systems (Figs. 9, 10 and 11). Notably, the dose distribution remained symmetric between the right and left glands. Because dynamic beam modulation in units B and C is restricted to the posterior cervical spine region (spanning a 40º to 60º arc), it does not affect the lateral transit of the primary beam over the parotid glands. Therefore, the pronounced dose amplitude is strictly governed by the inherent operational protocols of each unit. Unit operating at higher nominal tube voltages (e.g., unit C at 67–73 kV) delivered substantially higher doses to these lateral structures compared to units utilizing lower baseline potentials (e.g., unit A at 65–67 kV), independent of the sliding rotation axis mechanics.

#### Brain

When analysing organs located predominantly outside the primary field and exposed mainly to scattered radiation, particularly the brain, a similar dosimetric pattern was observed between sexes. However, the absorbed dose in the female phantom was 5.2% to 7.5% higher compared to the male (Table 6). Absolute brain doses remained consistently below 50 µGy. This slight increase in the female MRCP is driven by anatomical factors [15], specifically the smaller craniofacial dimensions, which position the base of the brain geometrically closer to the upper edge of the primary X-ray beam. Furthermore, the overall dose magnitude was strictly dependent on the specific unit protocol. These dosimetric trends and variations are in agreement with the findings reported by Li et al. [11].

#### Extrathoracic lymph nodes

Energy deposition in the extrathoracic (ET) lymph nodes did not exhibit a simple linear correlation with the increase in tube voltage (kV), revealing instead a strong dependence on the operational characteristics of each panoramic unit. In Unit A, which operates with a fixed isocentric rotation without modulation, increasing the voltage from 65 to 67 kV resulted in a slight dose reduction in both the female (330 to 322 µGy) and male (371 to 361 µGy) phantoms, reflecting automatic exposure compensation via mAs reduction. In contrast, systems featuring sliding axes and exposure modulation (Units B and C) demonstrated a direct dose escalation with increasing potential. In Unit B, the dose increased for both sexes (from 470 to 530 µGy in the female phantom, and from 531 to 588 µGy in the male phantom when moving from 63 to 69 kV). Unit C exhibited the most pronounced increase, reaching peaks of 783 µGy in the female phantom and 885 µGy in the male phantom at a tube voltage of 73 kV (Figs. 9, 10 and 11).

Despite these fluctuations dictated by unit-specific parameters, a dosimetric trend regarding patient sex was observed: the male phantom systematically absorbed 12% to 13% more dose in the ET lymph nodes than the female model across all systems (Table 6). During the panoramic exposure, the X-ray source orbits the posterior aspect of the patient, projecting the divergent beam through the cervical region towards the detector. Consequently, the lateral cervical chains, where the ET nodes are located, are directly intercepted by the primary beam. The 15-year-old male phantom possesses a greater cervical circumference, which exposes a broader cross-sectional area of this lymphatic network to the direct beam. This anatomical disparity accounts for the higher energy deposition compared to the more compact female cervical structure.

### Effect of beam modulation on absorbed dose across different units

A comprehensive comparison across the evaluated systems demonstrates that the influence of the operational protocol—specifically beam-emission technology and X-ray energy—outweighs anatomical sex differences regarding the absolute dose (Figs. 10 and 11). The most substantial dosimetric variations were observed in Unit B, likely driven by its wider energy interval between protocols (6.0 kV) combined with dynamic dose-modulation, which markedly alters the incident photon spectrum compared to the fixed-parameter approach of Unit A. Furthermore, while higher-energy beams (such as the 73 kV protocol in Unit C) exhibit greater penetrability and theoretically reduce the relative impact of cortical bony shielding for deep-seated organs, they concurrently escalate the overall energy deposition across all tissues (Table 6).

The greater sex-dependent dosimetric deviations observed with Unit C arise from its wider kV modulation range, which elevates the photon spectrum to peaks of up to 84 kV during the passage through the cervical spine, hardening the photon spectrum and reducing the relative contribution of the photoelectric effect (∝ Z³/E³), in favour of Compton scatter attenuation, which depends primarily on tissue thickness. Under these conditions, the difference in cervical circumference between the male and female phantoms becomes the dominant factor in differential attenuation, amplifying sex-related deviations in structures along the primary beam path, particularly in the oral mucosa of the lips and chin, where the deviation reaches − 24.1% with Unit C (73 kV nominal), compared with − 3.6% with Unit A (67 kV) and − 14.3% with Unit B (69 kV). Although the nominal kV variation between protocols is identical (6 kV) for Units B and C, the absolute peak voltages reached during modulation (B: 63 → 70 kV and 69 → 77 kV; C: 68 → 78 kV and 73 → 84 kV) result in physically distinct attenuation conditions, thereby explaining the greater sex-dependent dosimetric sensitivity observed with the higher-energy system.

Beyond sex-dependent differences, the dose magnitude observed between the two protocols of each modulated unit also reflects more than a simple 6 kV spectral shift. In Units B and C, the nominal tube voltage represents only the baseline value; during modulation, the kV rises substantially above this level, reaching absolute peak voltages of approximately 70 and 77 kV for Unit B, and 78 and 84 kV for Unit C, at the lower and higher nominal settings, respectively. This difference in peak intensity is directly captured by the conversion factors in Table 5: for Unit B, the factor increases from 5.03 to 5.68 between protocols, whereas for Unit C it increases from 5.18 to 9.38, indicating that the experimentally delivered air kerma–area product at 73 kV is approximately 1.8 times higher than at 67 kV. Consequently, the dose differences between protocols in Figs. 10 and 11 reflect the combined effect of a harder photon spectrum and a substantially greater total beam output, rather than a marginal spectral change.

Therefore, a clear dosimetric hierarchy emerges from this study: although the patient’s anatomy primarily determines the relative distribution of the dose (i.e., which specific structures intercept more or less radiation due to geometry and shielding), the unit’s operational protocol and beam energy determine the absolute magnitude of the exposure. From a radiological protection perspective, this confirms that dynamic exposure parameters are the primary determinants of patient risk in adolescent panoramic radiography.

**Table 6:** Relative percentage difference in absorbed dose: male regarding female

Organs	Unit A		Unit B		Unit C	
	% Ratio (Dose_Male_-Dose_Female_/Dose_Female_)					
	65 kV	67 kV	63 kV	69 kV	67 kV	73 kV
Thyroid	14.1%	14.5%	14.7%	15.5%	16.1%	16.1%
Salivary glands right	−0.5%	−0.6%	−0.2%	−0.6%	1.5%	0.7%
Salivary glands left	−0.7%	−0.9%	−1.2%	−1.4%	−1.9%	−1.9%
Oral Muc tongue	−10.7%	−14.5%	−13.6%	−14.4%	−12.8%	−12.5%
Oral Muc floor of mouth	−3.6%	−9.3%	−24.1%	−19.5%	−16.2%	−16.6%
Oral Muc lips and chin	−12.7%	−3.6%	−6.3%	−14.3%	−4.8%	−24.1%
Brain	−5.4%	−5.3%	−7.5%	−5.9%	−6.1%	−5.2%
Tongue, upper	−18.8%	−18.4%	−10.4%	−9.4%	−12.7%	−12.0%
Tongue, lower	−11.3%	−10.9%	−11.8%	−10.9%	−10.5%	−9.9%
Lymph nodes-Head	−26.5%	−26.7%	−27.8%	−26.9%	−26.7%	−25.2%
Lymph nodes-ET	12.6%	12.3%	12.7%	12.3%	13.0%	13.0%

### Effects of kV modulation on absorbed dose

To understand the impact of kV modulation on dose distribution, the analysis was performed on a case-by-case basis by combining the data in Table 7 with Figs. 10 and 11. In all units, the total rotation arc was 240° (from − 120° to + 120°), but only Units B and C apply kV modulation within a restricted angular sector. In Unit B, this modulation occurs approximately between − 30° and + 30°, whereas in Unit C it is concentrated between − 20° and + 20°, corresponding to the passage of the beam through the posterior portion of the skull.

Within the angular range in which modulation occurs, the dosimetric response no longer follows the pattern observed for Unit A and instead reflects the specific characteristics of each system’s modulation algorithm. An illustrative example is the “Oral Muc lips and chin” region. In Unit A, without modulation, increasing the tube potential from 65 to 67 kV reduces by approximately 9.1% points the relative dose difference for the female phantom compared with the male phantom (from − 12.7% to − 3.6% in Table 7). In Units B and C, however, this behaviour is reversed: in Unit B, when the tube potential is increased from 63 to 69 kV, the relative dose difference for the female phantom compared with the male phantom changes from − 6.3% to − 14.3%, that is, it increases by 8.0% points. In Unit C, the difference changes from − 4.8% to − 24.1%, corresponding to an increase of 19.3% points.

In contrast, the “Oral Muc floor of mouth” region exhibits a different behaviour. In Unit A, the relative dose difference for the female phantom compared with the male phantom is − 3.6% at 65 kV and − 9.3% at 67 kV, indicating that the female phantom consistently receives the higher dose and that the asymmetry between sexes becomes more pronounced as the tube potential increases. In Unit B, however, this relative difference decreases in magnitude with increasing kV, changing from − 24.1% to − 19.5%. In Unit C, in turn, the relative difference remains practically constant, at around − 16% for tube potentials of 67 and 73 kV. A plausible explanation is that, in Unit C, the angular sector (40°) subjected to kV modulation is narrower than in Unit B (60°), so that the contribution of the modulated sector to the mean dose in the floor‑of‑mouth mucosa becomes proportionally less relevant.

With regard to the brain, whose exposure arises predominantly from scattered radiation originating in the posterior region of the head, no change is observed in the relative dose difference between the phantoms for Unit A. In the angular range in which kV is increased to compensate for the higher bone attenuation in the cervical spine and cranial vault, modulation tends to reduce this difference between sexes in Units B and C, with decreases of approximately 1.6 and 0.9% points, respectively, when compared with the fixed‑beam scenario of Unit A.

Taken together, these findings indicate that kV modulation introduces an angular component dependent on local anatomy that can either accentuate or mitigate sex‑related dosimetric differences, depending on the organ’s position relative to the modulated sector. From a radioprotection perspective, this underscores the need to evaluate modulated protocols not only in terms of overall dose, but also in terms of the regional redistribution of dose in both superficial and deep tissues.

###  Integrating realistic physical modelling with ICRP 156 mesh phantoms

The interpretation of the dosimetric data presented in this study requires critical contextualisation against results available in the literature. To date, the great majority of dose estimates in panoramic radiography, including recent works such as those by Shatskiy [36] e Eren et al. [16], has been based on the use of codes such as PCXMC applied to stylised mathematical phantoms and/or voxel-based simulators. Although these models are valuable for reference estimates, the anatomical representation by geometric equations or discrete volumes imposes simplifications that do not fully capture the continuity and curvature of structures. By adopting mesh-type phantoms (ICRP 156) [15], this study goes beyond these approximations, providing a dose mapping that preserves surface geometry and the interconnection between neck organs, a particularly relevant aspect for small structures such as the thyroid and the salivary glands.

In addition to anatomical fidelity, our data incorporate the physical modelling of the radiation beam via PHITS. While common practice in simplified Monte Carlo simulations often assumes an idealised circular rotation trajectory with a single centre and fixed kV [6, 16, 36], the clinical reality of modern panoramic unit is mechanically more complex. The innovation of this work lies in implementing a realistic rotation path, approximated by an elliptical arc with a sliding rotation centre, combined with kV variation, which ensures that the simulated dose deposition reproduces the photon fluence during acquisition with greater fidelity. Thus, the absorbed doses presented herein represent not only idealised scenarios but physically more consistent approximations of the interaction of radiation with matter under dynamic conditions similar to clinical settings.

Therefore, the mesh-type phantom approach integrated with PHITS is not merely a technical refinement but a dosimetric necessity for the 15-year-old age group. In a transitional anatomy, where the distance between the mandible (the primary target) and the thyroid (the organ at risk) is reduced compared with adults, it becomes necessary to consider and discuss the components of scattered radiation dose. Thus, by reducing the uncertainties associated with voxel discretisation, this study suggests that radiological protection for adolescents should be grounded in models capable of capturing the biological and physical complexity of the examination, thereby increasing the level of confidence for clinical decision-making and protocol optimisation.

### The advantage of the mesh-type model over traditional physical dosimetry

The validation of computational models requires comparison with experimental physical dosimetry (TLD, OSL, MOSFET) [9, 11, 33, 37–39], which has historically underpinned the development of radiological protection standards. However, when we compare the data obtained with the ICRP 156 [15] mesh-type phantom with classical experimental studies such as those by Granlund et al. [4] and Heiden et al. [9], it becomes evident that the computational approach mitigates some of the intrinsic limitations of physical methods. Although these studies are fundamental, they rely on interpolating doses measured at discrete points, whereas the mesh-type model enables calculation of energy deposition in entire organs in a continuous manner, providing a more comprehensive volumetric estimate of absorbed dose.

This methodological distinction is particularly critical when assessing the salivary glands. When we contrast our data with those of Lee et al. [37], who used a phantom with dimensions similar to those of an adolescent (155 cm, 50 kg) and reported point doses on the order of 80 µGy, the ICRP 156 mesh-type model offers the additional advantage of enabling a fully volumetric assessment (Table 7). In this case, it was possible to estimate the dose not only in the parotid region, but also in the submandibular gland and in the oral mucosa (49.30–59.94 µGy), tissues that are often omitted or underestimated in physical dosimetry owing to instrumental access limitations.

Beyond volumetric accuracy, our results also corroborate the importance of sexual dimorphism in dosimetry (Table 7), as experimentally demonstrated by Li et al. [11]. That study showed that women and, by extension, individuals of shorter stature such as adolescents, tend to receive higher absorbed doses in cervical organs due to their smaller body mass and the proximity of these organs to the primary beam. Our findings confirmed this anatomical trend, particularly in the oral mucosa and brain, where the female phantom exhibited consistently higher doses than the male one (e.g., oral mucosa: female 55.48 µGy vs. male 49.30 µGy in Unit A). These results highlight that radiological protection strategies cannot follow a generic approach. Computational dosimetry studies should account for anatomical differences in organ mass and positioning, a feature accurately represented by Mesh-type phantoms, which also eliminate positioning errors that can significantly affect experimental dosimetry, as previously reported by Ahmadi et al. [38].

It is important to emphasise that, although the ICRP 156 phantom represents a high-fidelity “reference geometry”, clinical practice involves multiple variables. Dorsey et al. [39], used OSL dosimeters in paediatric phantoms and showed that the absorbed dose from an examination can vary substantially, potentially doubling or even tripling, depending on the brand and model of the unit. Thus, our results provide an anatomical reference parameter, but the final stage of clinical optimisation must be tailored to the specific imaging system.

Furthermore, the inherent limitations of experimental dosimetry result in substantial variability in the tissue and organ doses reported in the literature. As summarised in Table 6, these range from 40 to 201.3 µGy in the thyroid, 55 to 3397 µGy in the salivary glands, and 33 to 100 µGy in the brain.

By contrast, the doses calculated in this study are considerably lower (14.64 to 46.76 µGy in the thyroid and 4.20 to 12.62 µGy in the brain), as they derive from volumetric integration rather than the point measurements typical of experimental approaches. For the salivary glands, although our values (285.17–637.29 µGy) fall within the broad literature range (55–3397 µGy), they remain well below the reported upper limits.

Nevertheless, even when considering these variables, panoramic radiography remains an essential examination for the diagnosis and treatment planning of dental conditions in adolescentes [40]. Furthermore, this modality provides comprehensive imaging of the maxillomandibular complex at a relatively low radiation dose, especially when compared to other radiological techniques such as cone-beam computed tomography (CBCT) or full-mouth intraoral radiography.

**Table 7:** Comparative analysis of organ absorbed doses obtained using 15-year-old male and female mesh-type phantoms relative to experimental results reported in the literature (values in µGy)

Organ/Tissue	Li et al. [11]		Lee et al. [37]	Ahmadi et al. [38]	Heiden et al. [9]	Benchimol et al. [33]	Granlund et al. [4]	Dorsey et al. [39]	Study present ^1^					
Method	TLD-100 H (High Sens.)		RPL e OSL	TLD-100	TLD-100	MOSFET	TLD	OSL (Nanodot)	Mesh-type	Mesh-type	Mesh-type	Mesh-type	Mesh-type	Mesh-type
Sex	Male²	Female²	Female³	Male^4^	Male^5^	Male^4^	Male^5^	Male^6^	Male (A)	Female (A)	Male (B)	Female (B)	Male (C)	Female (C)
Thyroid	54.31	63.90	60–75	46.75	230	40	40–126	101.7–201.6	16.70–16.83	14.64/14.70	23.13/28.29	20.15/24.48	24.71/46.76	21.27/40.25
Salivary Glands	113.30	148.55	55–110	3397.48	520	890	869–2887	570.7–1089.9	292.59–285.17	294.35/287.30	316.51/352.90	316.69/356.52	351.74/633.42	352.50/637.29
Brain	33.10	29.59	*-*	*-*	*-*	100	5–26	10.1–20.4	4.19–4.18	4.43/4.41	6.18/7.26	6.67/7.70	6.44/12.01	6.85/12.62

(A) Dabi Atlante/Eagle, (B) Instrumentarium/OP300 and (C) Vatech/PaX;

^1^The tube voltages for the units were as follows: unit A, 65 kV/67 kV; unit B, 67 kV/73 kV; and unit C, 63 kV/69 kV;

^2^Standard adult (CDP-R1 model, Chengdu Fangtuo, China) – male (1.70 m, 65 kg) and female (1.60 m, 58 kg)

^3^Standard woman (1.55 m, 50 kg);

^4^Alderson Rando (Standard adult);

^5^Twenty-year-old anthropomorphic phantom: Alderson Radiation Therapy (ART) phantom

^6^Phantom *CIRS ATOM Max Model 711 HN* (“Average Adult”)

### Study limitations

This study presents certain limitations that should be acknowledged. First, the Monte Carlo simulations assumed an ideal alignment and positioning of the 15-year-old MRCPs. In clinical practice, patient movement or positioning errors can alter the dose distribution, particularly in tightly collimated examinations like panoramic radiography. Second, while the inclusion of three different units provided insight into the impact of beam modulation, the results are specific to the exposure parameters and inherent filtration of these models and may not be universally extrapolated to all available unit. Finally, the simulated organ doses rely entirely on computational transport; although the primary beam was validated using experimental air kerma–area product measurements, in-phantom physical dosimetry was not performed to cross-verify the specific organ depositions. Finally, this study did not include simulations using adult mesh-type reference computational phantoms (ICRP Publication 145); a direct comparison between paediatric and adult dose estimates using the same methodology would further support the importance of age-specific dosimetric models and is proposed as a direction for future work.

## Conclusion

This study implemented the ICRP 156 mesh-type reference computational phantoms (MRCPs) coupled with the PHITS Monte Carlo code to assess organ doses in 15-year-old patients undergoing panoramic radiography. The methodology demonstrated that capturing the complex kinematics and specific beam modulation (tube voltage and dose rate) of different panoramic units is strictly necessary for an accurate dosimetric evaluation.

Findings indicate that the salivary glands and oral mucosa remain the most critically exposed organs. Doses reached up to 300 µGy in the salivary glands for unit with a fixed rotation axis and constant tube voltage, escalating to 650 µGy in systems utilizing a sliding rotation axis and modulated kV. Crucially, dynamic X-ray beam modulation alters the energy deposition pattern, revealing significant dose disparities between the male and female MRCPs specifically in anatomical regions subjected to active modulation, a variation not observed in non-modulated systems.

Ultimately, these results emphasize that 15-year-old patients should not be generalized dosimetrically as small adults; they require age-specific protocol optimization. The integration of highly realistic anatomical models with dynamic exposure parameters provides a robust framework for enforcing the ALARA principle in adolescent dental radiology.

## Data Availability

The datasets generated and analysed during the current study are available from the corresponding author on request.
